# Respiratory support with nasal high flow without supplemental oxygen in patients undergoing endoscopic retrograde cholangiopancreatography under moderate sedation: a prospective, randomized, single-center clinical trial

**DOI:** 10.1186/s12871-023-02125-w

**Published:** 2023-05-08

**Authors:** Hironori Sawase, Eisuke Ozawa, Hiroshi Yano, Taiga Ichinomiya, Rintaro Yano, Hisamitsu Miyaaki, Naohiro Komatsu, Takao Ayuse, Shinji Kurata, Shuntaro Sato, Maximilian Ichabod Pinkham, Stanislav Tatkov, Kazuto Ashizawa, Kazuyoshi Nagata, Kazuhiko Nakao

**Affiliations:** 1 Department of Clinical Oncology, Nagasaki University Graduate School of Biomedical Sciences, 1-7-1 Sakamoto, Nagasaki-Shi, Nagasaki, 852-8501 Japan; 2 Department of Gastroenterology and Hepatology, Nagasaki University Graduate School of Biomedical Sciences, 1-7-1 Sakamoto, Nagasaki-Shi, Nagasaki, 852-8501 Japan; 3 Department of Internal Medicine, National Hospital Organization Saga Hospital, 1-20-1 Hinode, Saga-Shi, Saga, 849-8577 Japan; 4 Clinical Research Center, Nagasaki University Hospital, 1-7-1 Sakamoto, Nagasaki-Shi, Nagasaki, 852-8501 Japan; 5 Department of Anesthesiology and Intensive Care Medicine, Nagasaki University Graduate School of Biomedical Sciences, 1-7-1 Sakamoto, Nagasaki-Shi, Nagasaki, 852-8501 Japan; 6 Department of Gastroenterology, Japan Community Healthcare Organization Isahaya General Hospital, 24-1 Eishohigashi-Machi, Isahaya-Shi, Nagasaki, 854-8501 Japan; 7 Department of Translational Medical Sciences, Division of Clinical Physiology, Nagasaki University Graduate School of Biomedical Sciences, 1-7-1 Sakamoto, Nagasaki-Shi, Nagasaki, 852-8501 Japan; 8 Fisher & Paykel Healthcare Ltd, 15 Maurice Paykel Place, East Tamaki, Auckland, 2013 New Zealand

**Keywords:** Nasal high flow, Endoscopic retrograde cholangiopancreatography, Sedation

## Abstract

**Background:**

Nasal high flow (NHF) may reduce hypoxia and hypercapnia during an endoscopic retrograde cholangiopancreatography (ERCP) procedure under sedation. The authors tested a hypothesis that NHF with room air during ERCP may prevent intraoperative hypercapnia and hypoxemia.

**Methods:**

In the prospective, open-label, single-center, clinical trial, 75 patients undergoing ERCP performed with moderate sedation were randomized to receive NHF with room air (40 to 60 L/min, *n* = 37) or low-flow O_2_ via a nasal cannula (1 to 2 L/min, *n* = 38) during the procedure. Transcutaneous CO_2_, peripheral arterial O_2_ saturation, a dose of administered sedative and analgesics were measured.

**Results:**

The primary outcome was the incidence of marked hypercapnia during an ERCP procedure under sedation observed in 1 patient (2.7%) in the NHF group and in 7 patients (18.4%) in the LFO group; statistical significance was found in the risk difference (-15.7%, 95% CI -29.1 – -2.4, *p* = 0.021) but not in the risk ratio (0.15, 95% CI 0.02 – 1.13, *p* = 0.066).

In secondary outcome analysis, the mean time-weighted total PtcCO_2_ was 47.2 mmHg in the NHF group and 48.2 mmHg in the LFO group, with no significant difference (-0.97, 95% CI -3.35 – 1.41, *p* = 0.421). The duration of hypercapnia did not differ markedly between the two groups either [median (range) in the NHF group: 7 (0 – 99); median (range) in the LFO group: 14.5 (0 – 206); *p* = 0.313] and the occurrence of hypoxemia during an ERCP procedure under sedation was observed in 3 patients (8.1%) in the NHF group and 2 patients (5.3%) in the LFO group, with no significant difference (*p* = 0.674).

**Conclusions:**

Respiratory support by NHF with room air did not reduce marked hypercapnia during ERCP under sedation relative to LFO. There was no significant difference in the occurrence of hypoxemia between the groups that may indicate an improvement of gas exchanges by NHF.

**Trial registration:**

jRCTs072190021.

The full date of first registration on jRCT: August 26, 2019.

## Background

For relatively invasive upper gastrointestinal endoscopy procedures, such as an endoscopic retrograde cholangiopancreatography (ERCP), moderate sedation is routinely used to reduce patient anxiety and can also improve the endoscopist’s satisfaction [1, 2]. However, it has been reported that the frequently used sedation during an ERCP is associated with the occurrence of deep sedation linked to possible respiratory depression at rates as high as 35% [3]. Respiratory complications that occur during sedation may have a higher risk of hypercapnia than hypoxemia [4–8]. During sedation, supplemental oxygen (O_2_) administered through a nasal cannula can maintain the peripheral arterial O_2_ saturation at a normal level; however, hypoventilation may still be sustained.

Nasal high flow (NHF) with or without supplemental O_2_ via a nasal cannula interface may provide respiratory support in patients under sedation. NHF is commonly used in patients with acute respiratory failure and there is a substantial interest in its use during a perioperative period and procedural sedation [9–11]. It improves respiratory function primarily by generating low-level positive airway pressure and reducing the re-breathing from anatomical dead space [12]. During sleep, NHF without supplemental O_2_ is capable of reducing the re-breathing of CO_2_ from anatomical dead space by 45% and lowering minute volume ventilation [13]. Several randomized controlled trials (RCTs) and a retrospective study of the application of NHF with supplemental O_2_ (50 to 100%) during ERCP with sedation have revealed the efficacy of NHF to preserve oxygenation and the prevention of hypercapnia [14–17].

The purpose of this clinical trial was to investigate NHF without supplemental O_2_ as a respiratory support during sedation in patients undergoing ERCP. Taken together, the authors hypothesized that nasal high flow (NHF) of air used during moderate sedation can improve PtcCO_2_ and maintain SpO_2_ > 90% without increasing FiO_2_ in the inspired gas, compared with low-flow O_2_ (LFO) supplementation.

## Methods

The study was conducted at Nagasaki University Hospital with the approval of Nagasaki University’s Clinical Research Review Board (`7180001) and written informed consent was obtained from all subjects participating in this study. This clinical trial was registered prior to patient enrollment in the Japan Registry of Clinical Trials (jRCTs072190021; Principal investigator: Takao Ayuse; Date of registration: August 26, 2019). The authors have previously reported the aims and protocol of the current study [18], which are briefly summarized below.

### Study design

The present clinical trial was a prospective, randomized, controlled, open-label, single-center investigation into the efficacy of NHF use in patients undergoing ERCP under intravenous anesthesia and was carried out and analyzed in accordance with Consolidated Standards of Reporting Trials (CONSORT) guidelines [19]. The trial comprised two groups of participants who were randomized 1:1 to receive either low-flow O_2_ via a nasal cannula (LFO) (1 to 2 L/min: FiO_2_ 0.22 to 0.28) or NHF at 40 to 60 L/min of room air (FiO_2_ 0.21) during the ERCP.

### Recruitment of participants

Participants were recruited from Nagasaki University Hospital and were required to provide written informed consent. The inclusion criteria were as follows: adult patients between the ages of 20 and 85 years who gave informed consent after being fully informed of all details of this study. The exclusion criteria were: 1) continuous administration of O_2_ by nasal cannula (home O_2_ therapy), 2) inability to breathe nasally, 3) use of antithrombotic drugs that could not be reduced or discontinued on the day before ERCP, 4) history of pneumothorax, 5) deemed inappropriate as a subject, 6) positive SARS-CoV-2 PCR test.

### Study protocol

All participants undergo an ERCP under sedation using midazolam (approximately 0.05 mg/kg) with pethidine hydrochloride (an initial dose of 35 mg) to maintain the appropriate level of sedation using the Ramsay scale (Ramsay score 4: patient exhibits brisk response to light glabellar tap or loud auditory stimulus; and Ramsay score 5: patient exhibits a sluggish response to light glabellar tap or loud auditory stimulus). The sedation level is evaluated by Ramsay score before the start of sedation, after administration of a sedative, before the insertion of a scope, and at regular intervals after the end of treatment. The sedation level during treatment is evaluated and recorded every 5 min in the same manner as other vital sign evaluations. The NHF is generated by the Airvo™ 2 (Fisher & Paykel Healthcare Ltd., Auckland, New Zealand) with heated humidified air at a flow rate of 40 to 60 L/min.

Sedation is performed by an endoscopist using midazolam (approximately 0.05 mg/kg) with pethidine hydrochloride (initial dose of 35 mg) under consultation with an anesthesiologist. Midazolam bolus or pethidine hydrochloride is administered when patients are suspected of experiencing excessive pain or discomfort. When additional intravenous pethidine hydrochloride is administered, the administration unit should be 17.5 mg.

Additional doses of midazolam or pethidine hydrochloride are administered at the discretion of the endoscopist, but basically pethidine hydrochloride supersedes midazolam. The depth of sedation is assessed using the Ramsay scale every 5 min, along with the total dose of sedatives and the amount of sedative drug and effective opioid analgesic drug administered and the timing of each amount.

The transcutaneous CO_2_ partial pressure (PtcCO_2_) value is continuously measured by TCM4 (Radiometer Inc., Japan) every 2 s and the measured PtcCO_2_ value is output and recorded on the patient’s monitor (Nihon Kohden, BSM-9101) every 1 min via a connected output cable for analog value along with vital signs data. At the same time, the endoscopist reconfirms the vital signs, drug dosage, administration time, etc. from the notes recorded by the nurses. PtcCO_2_ of 60 mmHg or higher is defined as marked hypercapnia. PtcCO_2_ of 50 mmHg or higher and lower than 60 mmHg is defined as moderate hypercapnia.

For the SpO_2_ as the secondary outcome, the authors use the occurrence of hypoxemia with SpO_2_ < 90%. Because most of the continuously measured SpO_2_ values in the patient’s monitor showed a normal value SpO_2_ of > 90%, all the values of SpO_2_ are not recorded on the data acquisition device (REDCap). Therefore, SpO_2_ < 90% is defined as an occurrence of hypoxemia during the procedure and only the exact values of the occurrence of hypoxemia with SpO_2_ < 90% are recorded in all cases during the procedure.

To ensure patients’ safety, rescue 50% supplemental O_2_ is added in the NHF group and the O_2_ flow rate increased to 4 to 5 L/min in the LFO group if necessary, in order to maintain an SpO_2_ value > 90% during an ERCP procedure under sedation in the case of the occurrence of hypoxemia, just after confirming the incident of hypoxemia as a primary outcome during the procedure.

### Outcomes

The primary endpoint:

- Incidence of marked hypercapnia with a maximum PtcCO_2_ of 60 mmHg or higher (equivalent to PaCO_2_ of 55 mmHg or higher) during ERCP with sedation.

The secondary endpoints were as follows:

1. Time-weighted total PtcCO_2_ during ERCP with sedation (Total PtcCO_2_ was calculated by totaling the PtcCO_2_ readings per minute and dividing this by the time measured.)
2. Duration of hypercapnia showing PtcCO_2_ of 50 mmHg or higher (equivalent to PaCO_2_ of 45 mmHg or higher) during ERCP with sedation
3. Incidence of hypoxemia with transcutaneous O_2_ saturation < 90% during ERCP with sedation
4. The depth of anesthesia using the Ramsay scale
5. The dose of anesthetic administered.

### Randomization and data management

The principal investigator or co-investigator enrolled patients using Research Electronic Data Capture (REDCap) after confirming their eligibility [20]. Participants were randomly assigned to the two groups (ratio of 1:1) using the block method. Data was collected and randomization was performed using REDCap. In line with the research plan, procedures were monitored to confirm that the study was conducted appropriately.

### Statistical analysis

As the authors have not found a previous study which evaluated the effect of NHF with air on PtcCO_2_, they plan to perform an exploratory clinical trial based on the feasibility of such a study. It was not possible to analyze or predict the required sample size in this clinical trial, so it was based on the estimated enrolment of 80 patients at Nagasaki University Hospital where approximately 250 ERCP procedures were performed under sedation over a one-year period. These 80 ERCP cases were assigned to two groups: the NHF group (40 cases) and the LFO group (40 cases).

The analysis population for the main analyses of this study is Full Analysis Set (FAS). FAS was defined as those subjects in the intention-to-treat for whom the efficacy endpoint, transcutaneous CO_2_ pressure, could be measured.

For efficacy analysis, the authors estimated risk ratios and 95% confidence intervals (CIs) for the occurrence of hypercapnia or marked hypercapnia by modified Poisson regression with body weight as an adjusted variable. In addition, risk differences were calculated by generalized regression analysis using the identity function. For hypoxia, risk ratios and risk differences were calculated without adjustment for body weight from the above model, because estimation was not unstable. The mean difference between the groups for PtcCO_2_ per unit time was also summarized and then compared using the Welch’s t-test. The Wilcoxon rank-sum test was used to analyze the Ramsay index. To calculate the relative risk, we adjusted the initial dose of sedative for body weight. Finally, the total dose of sedative was adjusted for body weight and risk ratios were calculated using Poisson regression with a logarithm of time as the offset term.

All statistical analyses were carried out using R, version 4.1.2. All *p* values were two sided, with *p* values less than 0.05 considered significant. Because of the potential for type I error due to multiple comparisons, findings for analyses of secondary endpoints should be interpreted as exploratory.

## Results

**Fig. 1:**
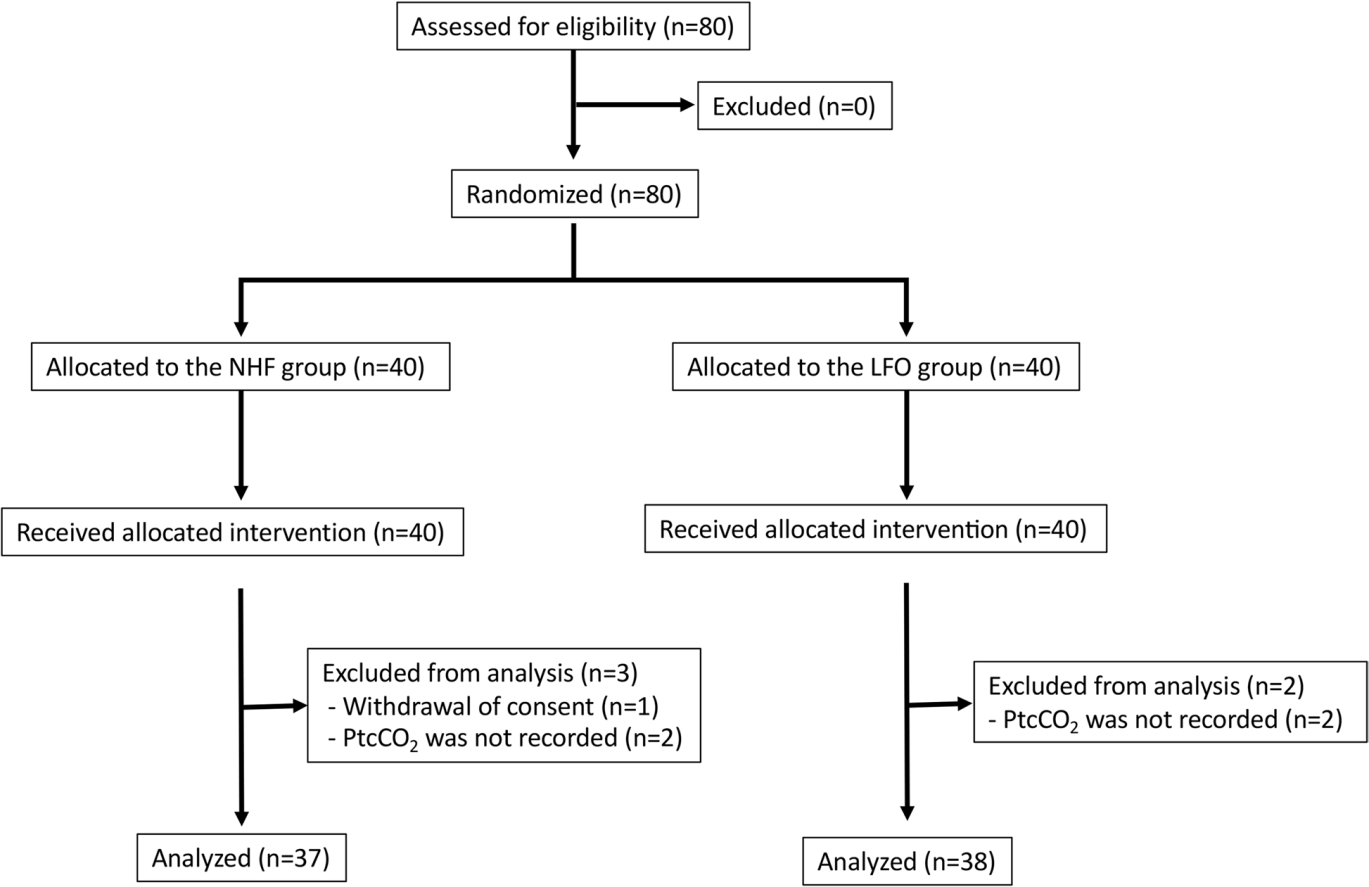
CONSORT flow chart. NHF: Nasal high flow, LFO: Low-flow oxygen via a nasal cannula, PtcCO_2_: Transcutaneous CO_2_ partial pressure

A total of 80 patients were enrolled and randomized to the NHF group (*n* = 40) or the LFO group (*n* = 40) from January 2020 to March 2021. As a result of exclusions, the number of patients analyzed in the NHF group was 37 and the number of patients analyzed in the LFO group was 38, as outlined in Fig. 1. The baseline characteristics of the patients and details of the scheduled procedures for each patient group, including the duration of the procedure and the presence of balloon-assisted enteroscopy (BAE) cases, are presented in Table 1. The duration of the procedure was 38.0 min (11–145) in the NHF group and 57.5 min (11–205) in the LFO group.

**Table 1:** The demographic data of patients in each group

	The NHF group *N* = 37	The LFO group *N* = 38
Age (years)	71.0 (43.0 to 83.0)	71.5 (35.0 to 83.0)
Sex, male	23 (62.0%)	23 (61.0%)
Weight (kg)	59.6 (31.3 to 79.2)	57.2 (39.6 to 104.3)
BMI (kg/m^2^)	22.4 (15.0 to 30.6)	21.9 (15.9 to 36.3)
Any tobacco use	21 (57.0%)	26 (68.0%)
FEV1.0% (%)	76.2 (63.1 to 100.0)	76.0 (53.2 to 90.9)
Details of treatment		
Detailed examination	10 (27.0%)	6 (15.8%)
Stent related (stenting or removal or replacement)	18 (48.6%)	20 (52.6%)
Endoscopic lithotripsy	9 (24.3%)	12 (32.6%)
Patients with surgically altered anatomy		
DG + Billroth II	0 (0%)	1 (2.6%)
DG or TG + Roux-en-Y	2 (5.4%)	4 (10.5%)
PD	1 (2.7%)	3 (7.9%)
The presence of BAE-ERCP	3 (8.1%)	7 (18.4%)

Values are* n* (%) or median (minimum to maximum)

*LFO* Low-flow oxygen via a nasal cannula

*BMI* Body mass index, *FEV1.0%* Forced expiratory volume % in one second

*DG* Distal gastrectomy, *TG* Total gastrectomy, *PD* Pancreaticoduodenectomy, *BAE* Balloon-assisted enteroscopy

In primary outcome analysis, the incidence of marked hypercapnia during an ERCP procedure under sedation was observed in 1 patient (2.7%) in the NHF group and in 7 patients (18.4%) in the LFO group; statistical significance was found in the risk difference (risk difference: -15.7%, 95% CI -29.1 – -2.4, *p* = 0.021; risk ratio: 0.15, 95% CI 0.02 – 1.13, *p* = 0.066) (see Table 2).

**Table 2:** Primary and secondary endpoints

	The NHF group *N* = 37	The LFO group *N* = 38	Association measure	Estimate (95% CI)	*p* value
Incident of marked hypercapnia (the proportion of patients experiencing a PtCO_2_ ≥ 60 mmHg)	2.7% (1/37)	18.4% (7/38)	Risk ratio^a^	0.15 (0.02 to 1.13)	0.066
			Risk difference (%)^b^	-15.7 (-29.1 to -2.4)	0.021
Time-weighted average for PtcCO_2_ (mmHg/min)^c^	47.2 ± 4.6	48.2 ± 5.7	Mean difference	-0.97 (-3.35 to 1.41)	0.421
Duration of hypercapnia (min)^d^ (PtCO_2_ ≥ 50 mmHg)	7 (0 to 99)	14.5 (0 to 206)			0.313
Incident of hypoxia (SpO_2_ ≦ 90%) (the proportion of patients experiencing an SpO_2_ ≦ 90%)	8.1% (3/37)	5.3% (2/38)	Risk ratio^e^	1.54 (0.27 to 8.70)	0.62
			Risk difference (%)^f^	2.8 (-8.5 to 14.2)	0.62
Hypercapnia (PtCO_2_ ≥ 50 mmHg)	56.8% (21 /37)	63.2% (24/38)	Risk ratio^a^	0.91 (0.63 to 1.31)	0.605
			Risk difference (%)^b^	-7.5 (-29.0 to 14.0)	0.495
Ramsay score at start of procedure^g^	5 (4 to 6)	5 (4 to 6)			0.488
Initial dose of pethidine hydrochloride (mg)	35 (0 to 35)	35 (0 to 35)	Relative Risk^h^	0.99 (0.91 to 1.07)	0.761
Initial dose of midazolam (mg)	3 (2 to 5)	3 (2 to 6)	Relative Risk^h^	0.99 (0.77 to 1.26)	0.904
Total dose of pethidine hydrochloride (mg)	35 (0 to 105)	70 (0 to 175)	Rate ratio^i^	0.96 (0.91 to 1.02)	0.177
Total dose of midazolam (mg)	6.0 (2 to 20)	7.5 (3 to 35)	Rate ratio^i^	1.00 (0.85 to 1.17)	0.967

Values are *n* (%), mean ± SD or median (minimum to maximum). *LFO* Low-flow oxygen via a nasal cannula, *PtcCO*_*2*_ Transcutaneous CO_2_ partial pressure, *SpO*_*2*_ Transcutaneous oxygen saturation

^a^Modified Poisson regression with body weight as an adjusted variable

^b^Difference in risk per 100 population. Generalized linear model with body weight as an adjusted variable

^c^Analysis duration: 0 to 120 min Welch’s t-test

^d^Fisher exact test

^e^Modified Poisson regression

^f^ Generalized linear model

^g^Wilcoxon’s rank-sum test

^h^Poisson regression with body weight as an adjusted variable

^i^Poisson regression, log (time) as offset term, with body weight as an adjusted variable

**Fig. 2:**
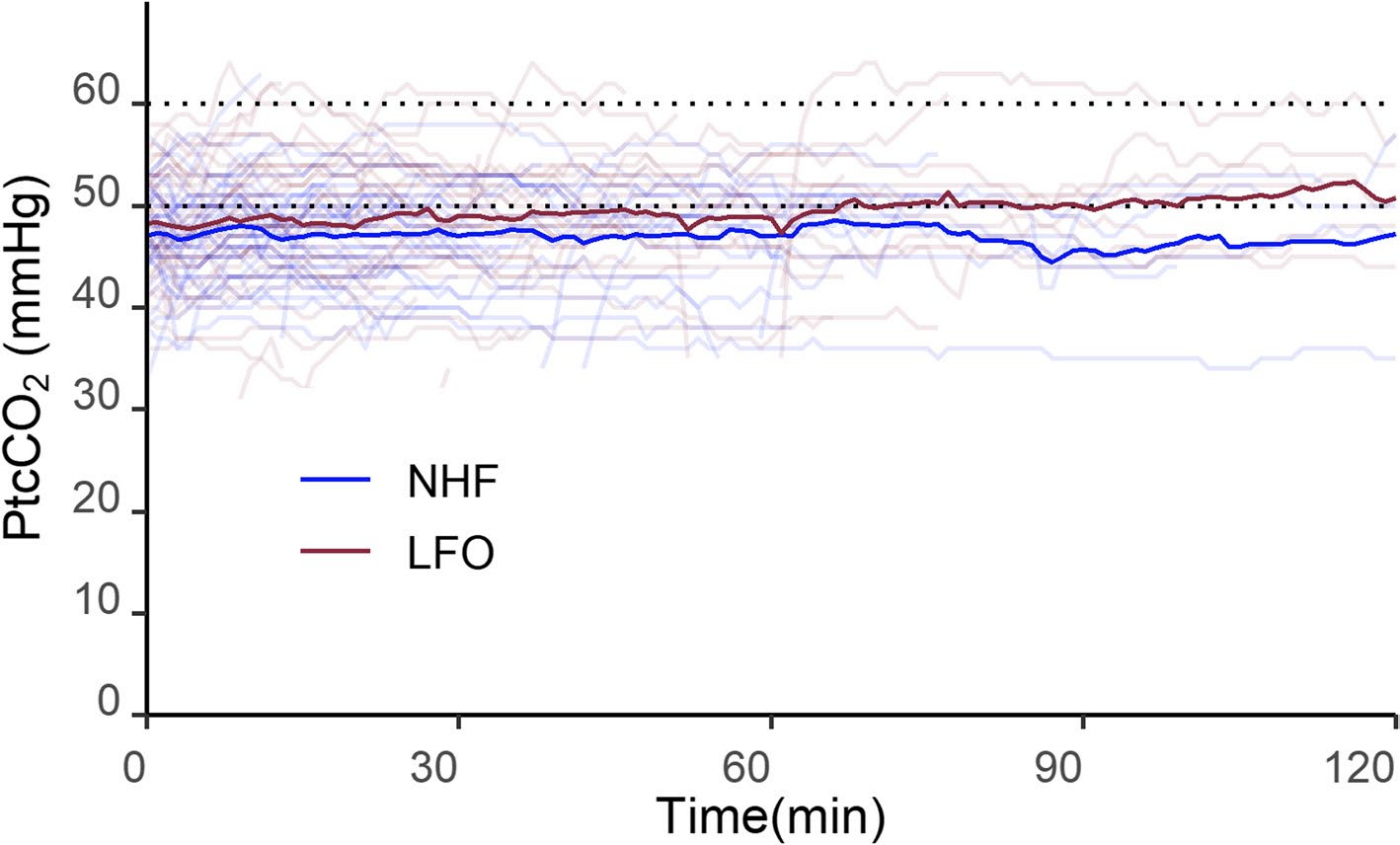
Sequence of PtcCO_2_ value of each group throughout this study. PtcCO_2_ values are presented as means. LFO: Low-flow oxygen via a nasal cannula

In secondary outcome analysis, the mean (SD) total PtcCO_2_ per unit time was 47.2 mmHg (4.6) in the NHF group and 48.2 mmHg (5.7) in the LFO group, with no significant difference (-0.97, 95% CI -3.35 – 1.41, *p* = 0.421). The duration of hypercapnia did not differ markedly between the two groups either [median (range) in the NHF group: 7 (0 – 99); median (range) in the LFO group: 14.5 (0 – 206); *p* = 0.313]. Figure 2 shows the timeline sequence of PtcCO_2_ value for each group and suggests that although there was no statistically significant difference, this might indicate a tendency for the changes in PtcCO_2_ to differ between the two groups over time.

The incidence of hypoxemia during an ERCP procedure under sedation was observed in 3 patients (8.1%) in the NHF group and 2 patients (5.3%) in the LFO group, with no significant difference (risk difference: -2.8%, 95% CI -8.5 – 14.2; risk ratio: 1.54, 95% CI 0.27 – 8.70; *p* = 0.62).

The depth of sedation (Ramsay score) was not significantly different between the two groups [median (range) for the NHF group: 5 (4 – 6); median (range) for the LFO group: 5 (4 – 6); *p* = 0.488]. In regard to the anesthesia dose, there was no difference between the initial and total dose of midazolam and the initial dose of pethidine hydrochloride. The total dose of pethidine hydrochloride (mg) was not different in the NHF group compared with the LFO group [median (range) for the NHF group: 35 (0 – 105); median (range) for the LFO group: 70 (0 – 175); *p* = 0.177]. There was no occurrence of any adverse events [18].

## Discussion

The main findings of this clinical trial are: 1) there was no significant difference in the incidence of marked hypercapnia with PtCO_2_ ≥ 60 mmHg and in the time-weighted total PtcCO_2_ during ERCP with sedation and in the hypoxemia with SpO_2_ ≦ 90% during ERCP with sedation between the NHF-with-air group and the LFO group; 2) there was no significant difference in the occurrence of hypoxemia between the groups that may indicate an improvement of gas exchange by NHF without supplemental O_2_.

### Effect of NHF on arterial and tissue carbon dioxide

In this clinical trial, there was no significant difference in the occurrence of marked hypercapnia between the NHF group and the LFO group with limited sample size. Although there was no statistically significant difference, this might indicate a tendency for the changes in PtcCO_2_ to differ between the two groups over time (Table 2). This finding is consistent with a recent study of 126 adults undergoing elective cardiac implantable electronic device procedures under sedation by Conway et al. [21] which reported that the difference in PtcCO_2_ was 0 kPa between NHF at 50 L/min and FiO_2_ of 0.50 or face-mask O_2_ at 8 L/min. Vijitpavan A et al. [22] suggested that the use of NHF in patients undergoing endovascular surgery under deep sedation reduced desaturation events when compared with conventional O_2_ via nasal cannula with no difference in the level of PaCO_2_ in arterial blood gas analyses (42.48 in conventional nasal canula vs. 44.8 in NHF; *p* = 0.107). Higuchi H. et al. [23] suggested that the PaCO_2_ was 50.1 ± 6.0 mmHg in the standard nasal cannula group and 47.6 ± 4.8 mmHg in the NHF group (flow 40 L/min, 40% O_2_).

### Effect of NHF on oxygenation

In this clinical trial, there was no significant difference in the incidence of hypoxemia between the two groups. NHF without supplemental O_2_ was chosen to verify whether the respiratory support could improve gas exchange and normalize both O_2_ and CO_2_ during hypoventilation caused by sedation. It was found that O_2_ saturation could be maintained with NHF with room air during an ERCP procedure under moderate sedation. Although a limited number of studies in the past have shown that hypoxemia could be attenuated with the use of NHF during endoscopy in ERCP procedures [9, 17, 24, 25], most of these studies also provided supplemental O_2_ (50% to 100%) and in the current study supplemental O_2_ was not required. Conway et al. [21] reported that the odds ratio for O_2_ desaturation for the NHF with 50% O_2_ supplement group was 1:2. Nay et al. [26] suggested that a decrease in SpO_2_ < 92% occurred in 9.4% for the NHF with 50% O_2_ supplement group and 33.5% for the standard O_2_ groups in 379 patients undergoing gastrointestinal endoscopy under deep sedation. Lin et al. [25] reported that NHF with 100% O_2_ supplement also decreased the incidence of hypoxia from 8.4% in the nasal cannula group to 0% (*P* < 0.001) in the NHF group. Mazzeffi MA et al. [27] reported that high-flow nasal cannula O_2_ significantly reduced the incidence of hypoxemia events from 33.1% to 21.2% (*P* = 0.03) in a recent clinical study of 262 esophagogastroduodenoscopy patients.

The study data adds to these earlier findings, suggesting that the positive airway pressure and reduction of dead space provided by NHF can be effective in maintaining oxygenation without supplemental O_2_ during ERCP procedures under moderate sedation compared to LFO.

## Limitations

There are several limitations in this clinical trial. First, the authors’ findings should be carefully generalized because of the limited number of patients enrolled and given the heterogeneous nature of the Japanese patients taking part in this study. Although the administration of midazolam or pethidine hydrochloride was based on the arbitrary evaluation by endoscopists in a single research center, it is not possible to indicate standardized results in this study.

Second, the average BMI (kg/m^2^) of the enrolled Japanese patients was 22.4 (15.0 –30.6) for the NHF group and 21.9 (15.9 – 36.3) for the LFO group, which is less than 25 of the obesity criteria. However, previous studies testing the effects of NHF on obese patients have revealed that NHF cannot reduce the incidence of desaturation compared to standard nasal cannula or face mask. Riccio CA et al. [10] suggested that the desaturation rates in the high-flow nasal cannula group (*n* = 28, BMI: 48 kg/m^2^) (39.3%) and the standard nasal cannula group (*n* = 31, BMI: 49 kg/m^2^) (45.2%) were not significantly different (*p* = 0.79). These researchers concluded that high- and low-flow O_2_ supplementation groups at similar FiO_2_ were not significantly different for the prevention of arterial O_2_ desaturation in morbidly obese patients undergoing propofol sedation for colonoscopy. Thiruvenkatarajan V et al. [15] reported that in high-risk obesity patients (BMI: 28.2 to 30.0 kg/m^2^) undergoing an ECRP procedure, O_2_ therapy with high-flow nasal O_2_also did not reduce the rate of hypoxemia or hypercapnia, compared with combined oral and nasal low-flow O_2_. Therefore, the authors of the present study consider that there is a need for further work to explore the optimal flow rate with O_2_ supplementation using NHF in high-risk patients for respiratory functions, including obesity and any respiratory complications, such as COPD.

Third, it should be noted that this trial was performed during moderate sedation. Deep sedation using another sedative agent, such as dexmedetomidine or anesthetics propofol, is now well accepted in managing ERCP. Vijitpavan et al. [22] suggested that the use of NHF in patients undergoing endovascular surgery under deep sedation using propofol reduced desaturation events greater than the use of a traditional nasal cannula. Therefore, further RCTs would be required to confirm the effects of NHF on respiratory management during ERCP under moderate to deep sedation.

Forth, masking was not possible due to the nature of the study.

Finally, in this study pethidine hydrochloride was administered when patients were suspected of experiencing excessive pain or discomfort without evaluating analgesia quantitatively using a VAS score as had been used in a previous study [28]. It might prove helpful to investigate the distinguished role of sedation and analgesia using any kind of analgesia rating score during this procedure, because analgesic can induce the respiratory depression rate and hypercapnia [29].

## Conclusion

Respiratory support by NHF with air did not reduce marked hypercapnia during ERCP under sedation relative to LFO. There was no significant difference in the occurrence of hypoxemia between the two groups that may indicate an improvement of gas exchange by NHF.

## Data Availability

The datasets used and/or analyzed during the current study are available on reasonable request from the author for correspondence.

## Abbreviations

BAE: Balloon-assisted enteroscopy
BMI: Body mass index
CI: Confidence interval
CO_2_: Carbon dioxide
CONSORT: Consolidated Standards of Reporting Trials
CRB: Clinical Research Review Board
DG: Distal gastrectomy
ERCP: Endoscopic retrograde cholangiopancreatography
FEV1.0%: Forced expiratory volume percentage in one second
FiO_2_: Fraction of inspired oxygen
jRCT: Japan Registry of Clinical Trials
LFO: Low-flow oxygen via a nasal cannula
NHF: Nasal high flow
PaCO_2_: Partial pressure of arterial carbon dioxide
PCR: Polymerase chain reaction
PD: Pancreaticoduodenectomy
PETCO_2_: Partial pressure of end-tidal carbon dioxide
PtcCO_2_: Transcutaneous CO_2_ partial pressure
RCT: Randomized controlled trial
REDCap: Research Electronic Data Capture
SD: Standard deviation
SpO_2_: Transcutaneous oxygen saturation
TG: Total gastrectomy
